# Gender and Racial Disparity Among Liver Transplantation Professionals: Report of a Global Survey

**DOI:** 10.3389/ti.2022.10506

**Published:** 2022-08-16

**Authors:** Victoria Aguilera, Oya Andacoglu, Claire Francoz, Gabriela Berlakovich, Sher-Lu Pai, Dieter Adelmann, Simantika Ghosh, Keri E. Lunsford, Martin Montenovo, Anna Mrzljak, Irene Scalera, Qinfen Xie, Chiara Becchetti, Marina Berenguer, Nazia Selzner

**Affiliations:** ^1^ Hepatology and Liver Transplant Unit, IIS La Fe and CIBER-EHD, Universitary and Politecnic Hospital La Fe, Valencia, Spain; ^2^ Department of Medicine, University of Valencia, Valencia, Spain; ^3^ Division of Transplantation, Department of Surgery, The University of Oklahoma College of Medicine, University of Oklahoma, Oklahoma City, OK, United States; ^4^ Transplant Surgery, International Liver Center, Istanbul, Turkey; ^5^ Liver Intensive Care Unit and Transplantation, Hepatology, Hospital Beaujon, Clichy, France; ^6^ Department of Transplant Surgery, Medical University of Vienna, Vienna, Austria; ^7^ Department of Anesthesiology and Perioperative Medicine, Mayo Clinic College of Medicine, Jacksonville, FL, United States; ^8^ Department of Anesthesia and Perioperative Care, University of California, San Francisco, San Francisco, CA, United States; ^9^ Department of Anesthesiology, Narayana Health, Narayana, India; ^10^ Department of Surgery, Division of Transplant and HPB Surgery, Rutgers New Jersey Medical School, Rutgers, The State University of New Jersey, Newark, NJ, United States; ^11^ Division of Hepatobiliary Surgery and Liver Transplant, Vanderbilt University Medical Center, Nashville, TN, United States; ^12^ Department of Gastroenterology and Hepatology, University Hospital Center Zagreb, Zagreb, Croatia; ^13^ Division of Hepatobiliary Surgery and Liver Transplant, University Hospital Policlinic of Bari, Bari, Italy; ^14^ Department of Hepatobiliary and Pancreatic Surgery, Shulan (Hangzhou) Hospital, Hangzhou, China; ^15^ University Clinic for Visceral Surgery and Medicine, Inselspital, University Hospital of Bern, Bern, Switzerland; ^16^ Multiorgan Transplant Program, University of Toronto, Toronto, ON, Canada

**Keywords:** liver transplantation, gender equality, leadership, women physicians, racial disparity

## Abstract

Equality, diversity, and inclusion (EDI) are fundamental principles. Little is known about the pattern of practice and perceptions of EDI among liver transplant (LT) providers. International Liver Transplant Society (ILTS) EDI Committee survey around topics related to discrimination, mentorship, and gender. Answers were collected and analyzed anonymously. Worldwide female leadership was also queried via publicly available data. The survey was e-mailed to 1312 ILTS members, 199 responses (40.7% female) were collected from 38 countries (15.2% response rate). Almost half were surgeons (45.7%), 27.6% hepatologists and 26.6% anesthetists. Among 856 LT programs worldwide, 8.2% of leadership positions were held by females, and 22% of division chiefs were female across all specialties. Sixty-eight of respondents (34.7%) reported some form of discrimination during training or at their current position, presumably related to gender/sexual orientation (20.6%), race/country of origin (25.2%) and others (7.1%). Less than half (43.7%) received mentorship when discrimination occurred. An association between female responses and discrimination, differences in compensation, and job promotion was observed. This survey reveals alarmingly high rate of experience with racial and gender disparity, lack of mentorship, and very low rates of female leadership in the LT field and calls to action to equity and inclusion.

## GRAPHICAL ABSTRACT

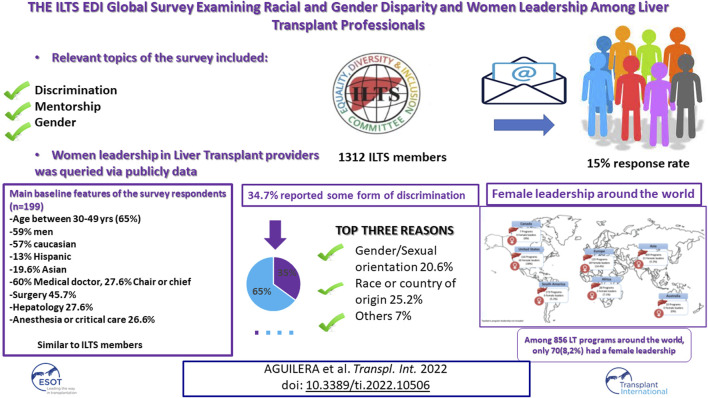

## Introduction

Recent years have seen an exponential growth of women and minority populations among medical trainees, and the increasing workforce diversity is gradually translating to medical and surgical subspecialties (1). Further workforce diversification occurs through immigration, allowing diverse groups to travel and build career paths beyond their countries of origin. As a result, medical professional environments are becoming increasingly multicultural, international, and diverse in terms of its specialists. However, according to most recent American Medical Association report, female physicians represent 36% of all physicians as of 2019 in the United States (2). Similarly, minority groups remain underrepresented both in training and leadership positions (1, 3–7).

Many transplant professionals face challenges related to gender or racial discrimination in their work environment or face inequalities such as access to leadership positions, professional promotion, and compensation. According to the recent report from Women in Transplantation Committee, a subcommittee of The Transplantation Society of Australia and New Zealand, women comprise more than half of the Australian medical doctoral graduates and early career researchers, however less than 20% of all academic medical professorial staff are women. The report also highlighted an even more striking gender disparity in composition of the professional workforce within the field of transplantation (5). Currently, there are no female heads of unit in any of the Australian or New Zealand transplanting centers. Similarly, the report on Spanish women hepatologists demonstrated that despite a slight predominance of women (*n* = 239, 56.3% vs. *n* = 184 men, 43.7%) in the workforce, only 15 (21.4%) high-ranking positions were held by women (6). Lack of diversity among transplant center leadership is of global concern.

The International Liver Transplant Society **(**ILTS) Equality, Diversity, and Inclusion (EDI) Committee is committed to promoting a supportive environment for all our members, irrespective of race, ethnicity, gender or sexual orientation. The key goal of the committee is to facilitate educational and professional development and to promote an inclusive working environment for all ILTS members. As a first step toward this mission, we sought to survey the ILTS members’ subjective experience with disparities or disadvantage related to gender, country of origin, and ethnicity. Respondents were asked to report their experiences with respect to leadership positions, promotion, mentorship opportunities and visibility at meetings within ILTS.

## Methods

The survey was designed and discussed with contribution by all EDI members to target the entire active ILTS membership. The primary objective of this survey was to delineate subjective opinions regarding disparities in professional training and practice. The second objective was to discern potential target areas for improvement in any of the possible discriminative issues. The survey included 19 questions related to age, gender identity, ethnicity, job position, and sex of the program leader. Further questions were related to perceived discrimination, disadvantage in mentorship opportunities, or parameters (Supplementary S1). The survey was distributed by ILTS to all members twice in November 2020 and September 2021. The responders were only able to complete the survey once and the questions were designed so only one answer were expected. All answers were collected anonymously. We collected demographic data of ILTS members including sex and age as well as location of practice and specialty as comparison group for the survey responders to determine whether the responders were representative of ILTS members.

We simultaneous conducted a search within the ILTS-EDI committee members’ networks of publicly available information using the GODT website (www.transplant-observatory.org). Specifically, each member collected data regarding leadership composition of centers within their countries in order expand the data on female leadership positions in transplant-related fields: Questions included: 1-Total number of liver transplant (LT) programs in the country; 2-Total number of females with the following titles: 1) LT medical director, 2) LT surgical director, and 3) LT program director/chief positions.

A descriptive analysis of variables was carried out. Categorical variables were reported as absolute frequencies. To explore the association with discrimination issues and gender, a Chi2 score was performed. A p-value below or equal to 0.05 was considered statistically significant. The study was exempted for REB University of Valencia (Supplementary S2).

## Results

The survey link was e-mailed to 1312 ILTS members and 199 responses were returned from members in 38 countries (15.2% response rate). Respondents were LT providers from Argentina, Australia, Austria, Belgium, Brazil, Canada, Chile, China, Colombia, Croatia, Egypt, France, Germany, India, Italy, Iran, Ireland, Japan, Mexico, Nepal, Netherlands, Nigeria, Norway, Pakistan, Philippines, Poland, Portugal, Puerto Rico, Russia, Spain, Singapore, Switzerland, Turkey, United Kingdom (UK), United Arab Emirates (UAE), United States of America (US), Ukraine, and Uruguay, which was similar to the ILTS membership geographic distribution (44% members from North America, 21% Europe 24% Asia and 11% others in ILTS members vs. 42%, 36%, 14% and 8.5%, respectively in survey respondents). Noticeably profile was similar between respondents and ILTS members, majority being younger than 50 years age in both groups (Tables 1, 2). Forty-one percent reported themselves as women and 59% as men. Regarding ethnicity distribution, most of the respondents were Caucasian (57.3%) followed by Asian (19.6%). Most respondents (*n* = 148, 74.4%) reported working in an academic hospital, 76.9% (*n* = 153) worked in large (≥50 LT per year) programs and 174 (87.4%) were in practice for 10 years or more. Almost one-third of the respondents (*n* = 55, 27.6%) were either chief of their division or occupied a chair position; while, only 14% of all ILTS members reported occupying a leadership role (see Table 1). Our respondent profile therefore included a relatively higher percentage of leaders compared to ILTS member profile. Surgeons were the most frequent respondents (*n* = 91, 45.7%), followed by hepatologist (*n* = 55, 27.6%), and the remaining were specialists of anesthesia/critical care (*n* = 53, 26.9%), pediatric medicine (*n* = 6, 3%) and other transplant-related specialties (pathology, education and research, organ allocation) (*n* = 19, 9.5%). This, again, is similar to ILTS membership with 46% surgeons, 28% anesthesia and 15% hepatologist 15%. However, our survey respondents had more hepatologists with more LT providers from Europe (See Supplementary Table).

**TABLE 1:** Baseline characteristics of the participants (only ILTS members) in the survey.

	N = 199
Age of respondents	
-<30 years	2 (1%)
-Between 30–39 years	51 (25.6%)
-Between 40–49 years	79 (39.7%)
-Between 50–59 years	45 (22.6%)
-Between 60–69 years	20 (10.1%)
->70 years	2 (1%)
Gender	
-Women	81 (40.7%)
-Men	118 (59.3%)
-Other	0 (0%)
Ethnicity	
-African or African American	4 (2%)
-Asian	39 (19.6%)
-Caucasian	114 (57.3%)
-Hispanic or Latino	26 (13.1%)
-Eastern	23 (11.5%)
-Other	2 (1%)
-Prefer no to answer	1 (0.5%)
Type of working place	
-Academic	148 (74.4%)
-Private	17 (8.5%)
-Government	29 (14.6%)
-Others	5 (2.5%)
Job Position	
-Chief or Chair	-55 (27.6%)
-Medical Doctor	120 (60.3%)
-Medical Doctor in Training	8 (4%)
-Researcher	7 (3.5%)
-Others	9 (4.5%)
Specialty^a^	
-Surgery	91 (45.7%)
-Hepatology	55 (27.6%)
-Anesthesia or Medical care	53 (26.6%)
-Pediatric Medicine	6 (3%)
-Others	19 (9.5%)
Gender of leadership position of the LT program	
In total	
-Men	155 (77.9%)
-Woman	44 (22.1%)
Surgery (*n* = 90)	
-Men	78 (85.7%)
-Women	13 (14.2%)
Hepatology (*n* = 54)	
-Men	44 (80%)
-Women	11 (20%)
Anesthesia (*n* = 53)	
-Men	36 (67.9%)
-Women	17 (32.1%)
Number of LT done in 1 year at each institution	
<50 LT	46 (23.1%)
-Between 50–100 LT	56 (28.1%)
-Between 100 and 200 LT	82 (41.2%)
->200 LT	15 (8.5%)

a Some reported more than one specialty.

**TABLE 2:** Discriminant issues among LT providers.

	N = 199
Any discrimination during your training or current position	
-Yes	69 (34.7%)
-No	130 (65.3%)
Type of discrimination^a^	
-Gender or sexual orientation	41 (20.6%)
-Race	27 (13.6%)
-Country of origin	23 (11.6%)
-Others	18 (9%)
-Prefer not to state	2 (1%)
-Religion	5 (2.5%)
Mentor Support in the event of discrimination^a^	
-Always	21 (10.6%)
-Usually	32 (16.1%)
-Sometimes	24 (12.1%)
-Rarely	34 (17.1%)
-Never	87 (43.7%)
Discrimination for job promotion	
-Yes	62 (31.1%)
-No	137 (68.8%)
Institutional support for maternity leave	
-Yes	81 (40.7%)
-No	29 (15.8%)
-Neither supportive or unsupportive	73 (39.9%)
Differences in compensations between men and women	
-Yes	34 (17.1%)
-No	165 (83%)
Disadvantages for participating in ILTS for country or language skills	
-Yes	37 (18.6%)
-No	162 (81.4%)

a Some reported more than one type of discrimination.

**FIGURE 1:**
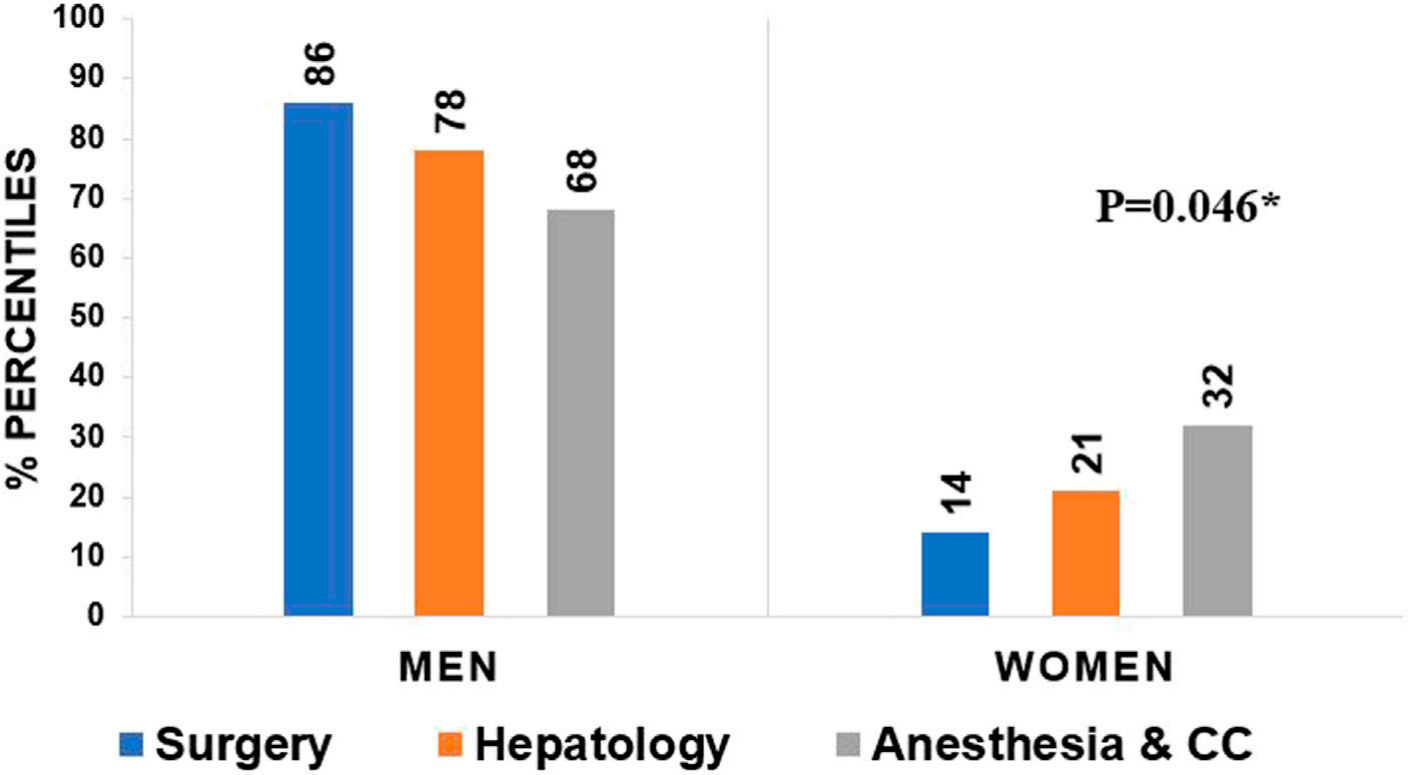
Leadership gender among specialties; there is a significant difference between men and women leadership position between different specialties (*p* = 0.046, Chi 2).

Only 22.1% (*n* = 44) respondents identified a woman department/division chief when all specialties were included. When we looked at the 3 largest subspecialties, there were only 13 (14.2%) female chiefs in transplant surgery, 11 in hepatology (20%) and 17 in anesthesia/critical care (32.1%). The incidence of female leadership was significantly different between specialties and the proportion of women was lower in surgery compared to hepatology or anesthesia/critical care (*p* = 0.046) (Figure 1).

Sixty-nine (34.7%) of respondents indicated some form of discrimination or disadvantages during training or current practice (Table 2). Top 3 reasons of presumed basis or discrimination were gender/sexual orientation (*n* = 41, 20.6%), race or country of origin (*n* = 50, 25.2%) and other (*n* = 14, 7.1%), which was described as “age, political view and pregnancy”. Eighty-seven professionals (*n* = 87, 43.7%) reported they never had a possibility to work with or receive support from a mentor during their training or career when a potential discrimination issue appeared. Sixty-two (31%) responded that they felt at disadvantage for a job promotion due to one of the above discriminations. Thirty-seven (18.6%) reported perceived disadvantage in relation to the country of origin or language skills in terms of participation at ILTS meetings or in leading collaborative projects. Although some forms of discrimination are not completely equal, we decided to combine gender and sexual orientation and race and country of origin due to the small number of respondents.

Less than half (40.7%) of survey respondents reported that they perceived their institution to be supportive of pregnancy. Thirty-four (17.1%) reported differences in compensations (salary, bonus, incentive payments, research stipends, honoraria, and distribution of profits to employees) between women and men within their workplace (Table 2).

We also compared answers between genders and 1) any type of discrimination, 2) discrimination in job promotion, 3) differences in compensation, 4) access to support for discrimination and 5) participation in ILTS. Women reported higher overall discrimination rates (*p* < 0.001), differences in compensation (*p* = 0.002), and discrimination in job promotion (*p* = 0.006) compared to men (Table 3).

**TABLE 3:** Responses based on gender and discrimination issues.

	No (%)	Yes (%)	P value
Suffer any type of discrimination			**<0.0001**
-Male (*n* = 118)	93	25 (21%)	
-Female (*n* = 81)	37	44 (54%)	
Differences in compensation			**0.002**
-Male (*n* = 118)	106	12 (10.1%)	
-Female (*n* = 81)	59	22 (27.1%)	
Discrimination in job promotion			**0.006**
-Male (*n* = 118)	90	28 (23.8%)	
-Female (*n* = 81)	47	34 (42%)	
Mentor support for discrimination			0.299
-Male (*n* = 118)	75	42 (36%)	
-Female (*n* = 81)	46	35 (43.2%)	
Participation in ILTS			0.445
-Male (*n* = 118)	94	24 (20.3%)	
-Female (*n* = 81)	68	13 (16%)	

Bold is for significant *p* values that is *p* < 0.05.

**FIGURE 2:**
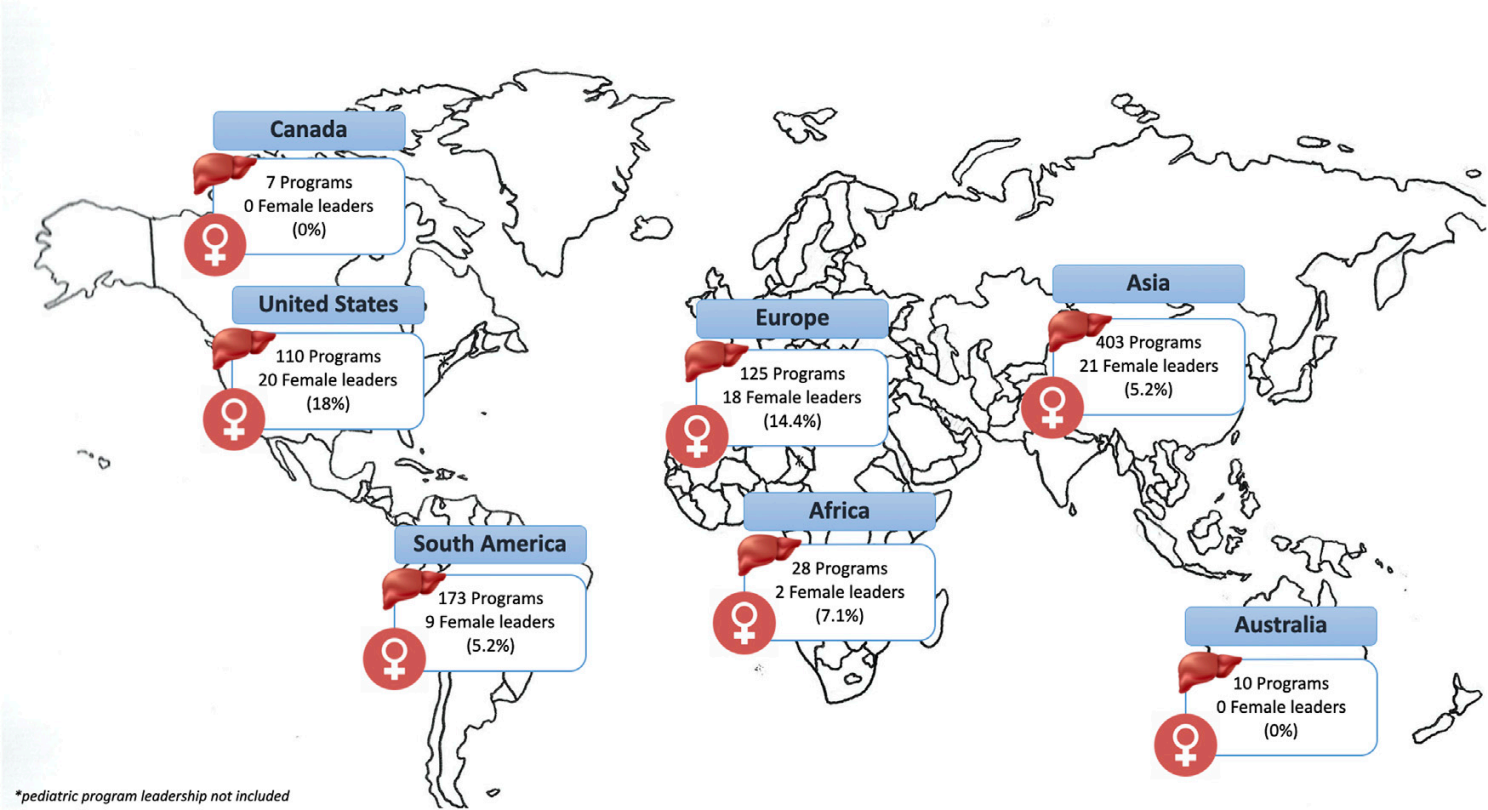
Global map of leadership composition in liver transplant. Data source: GODT website (www.transplant-observatory.org).

In addition, based on the individual network search, a global map of leadership composition in LT units was created (Figure 2). The search identified any female leaders in LT: medical or surgical directors, program director, or chief of LT program. The total number of total female leaders in each country was then divided by the total number of programs in the country. Female leadership positions composition was: North America, United States: 110 active LT programs with 20 (18.1%) female leadership, Canada: 7 LT programs with zero female leadership, South America: 173 LT programs, with 9 (5.2%) female leadership, Europe: 125 LT programs, with 18 (14.4%) female leadership, Asia: 403 LT programs, with 21 (5.2%) female leadership, Africa: 28 LT programs, with 2 (7.1%) female leadership, Australia: 10 LT programs, with zero female leadership positions. Out of 856 LT programs around the world, we were able to identify only 70 (8.2%) females in leadership positions.

## Discussion

ILTS represents a diverse society of liver transplant professionals including , surgeons, anesthesiologists, and research scientist, as well as other transplant professionals from around the globe. As such, ILTS is an ideal group for the study of discrimination and inequality differences prevalent in different medical specialties. Our current survey, although small in scale, represents the global distribution of ILTS members and reveals important findings that merit attention. First, even though 40% of the respondents identified as female between 40 and 59 years of age, only 22.1% of the group occupied a leadership position. Moreover, gender differences in leadership roles were most prominent amongst surgeons compared with hepatologist or anesthesia/critical care (14.2% vs. 20% vs. 32.1% respectively, *p* = 0.046). Unequal distribution of female professionals in leadership position has been previously reported across various fields in medicine (1, 3–7), and we now confirm a similar gender bias in liver transplantation. According to a recent report from United States, the female transplant workforce has increased from 3.7% in 1980 to 18.4% in 2010, however only 13.1% of practicing US transplant surgeons in that survey were female (8). It is highly likely that even fewer were practicing in the liver transplant area. It is difficult to ascertain with our results whether low women in LT is due to progression failure (i.e., women are not promoted to leadership roles after they are in practice) or if it is because there are too few women liver transplant surgeons. Historically women are minority among surgical specialties. Bingmer et al. reported women represents 32% of all surgical specialties according to the United States Census, Bureau of Labor Statistics and Association of American Colleges data between 2004–2018 (9). This imbalance appears compounded amongst liver transplant surgeons, and male representation remains disproportionately higher at advanced stages of the medical career (4, 7–8). Many actions need to be taken at the hospital/university level and within medical societies to encourage female participation as well as minorities to ensure adequate academic and clinical support in order to promote advancing in leadership positions in academic LT career paths. The first step is undoubtfully to gather granular data to fully understand the scope of the problem, as we have attempted to do with our survey. We will continue to create and work on various aspects of lower representation in LT field leadership.

These gender differences in leadership position among transplant centers are also highlighted by the world map representation (Figure 1). Out of 856 LT programs worldwide, only 8.2% of leadership positions were held by women. Most notable were the geographic differences in female leadership, with rates varying between 0–18% by continent. In addition, in the previous report by the EDI committee, lack of female leadership was similarly highlighted (Forthcoming: Accepted awaiting publication) (Figure 2).

Second or more strikingly, 34.7% of LT providers reported some form of discrimination, related to gender, sexual orientation, race, and country of origin. Again, female LT providers perceived higher discrimination rates (*p* < 0.0001). Although perceived discrimination is not an objective data, it is the legal term for discrimination and refers to individual's perception of negative attitude, judgement, or unfair treatment due to their specific characteristics such as gender, race, ethnicity, and social status. We acknowledge the subjective nature of self-reported discrimination; however we believe this is something we cannot change and yet as society and ILTS-EDI committee, we feel we need to investigate discrimination and all kind of disparities no matter how subjective it is. In addition, 43.7% reported they never had opportunity to work with or receive support from a mentor during training or current career, in relation to the discrimination issues. While great strides in gender equality have been made in the past 2 decades, further understanding of unintentional bias and micro aggressions are clearly necessary. Furthermore, career mentorship is one of the most important determinants of success in academic medicine and research. There is suggestion that successful mentoring programs should have intent, structure, process, resources and be evaluated (10). The ideal conductive system is not well defined, and some authors advocate for a more flexible or organic mentorship based on their objectives rather than over predefined or assigned mentor for success. Improved mentorship access, especially from those with similar racial, ethnic, or gender backgrounds, is critical in overcoming such disparities. While transplant societies are practicing mentor-mentee match programs in various fields, we propose every institution should initiate and foster mentor-mentee matches not only for trainees but also for staff or faculty. Along the same line, ILTS plans to promote mentorship among its members to rectify these situations, not only in terms of academic promotion, but also to identify and address any type of discrimination.

Another interesting information from the survey was that the support for maternity leave was only acknowledged by 44.2% of responders. The International Labor Office (ILO) highlights the importance to guarantee maternity leave as an essential means for preventing maternity from becoming a source of discrimination. According to 2017 report, the ILO’s estimates of the numbers covered reveal that 41% of employed women have a statutory right to maternity leave, and 34% of the totals are legally entitled to cash benefits during maternity leave^1^. Indeed, in medicine, some studies have reported higher wellbeing in residents with longer maternity leaves (11). Unfortunately, a recent study showed that perception about parental leave among surgical residents, including lack of knowledge regarding policies and lack of support from peers/faculty have an impact on considering surgery as a career (12). Our responses might reflect a complex mix of societal norms, cultural values, and government regulations. While we are aware there could be government or country specific regulations, we propose every center should review existing parental leave policies and should make the best possible effort to reform these into financially and structurally supportive policies.

Another remarkable issue is differences in promotion and compensation, such that 17.1% in our survey reported compensation differences between men and women.

Compensation differences has been reported in adjusted analyses and has potentially been linked to household responsibilities and childbearing, as well as difficulties in finding an effective mentor (13). In line with this finding, our survey showed that female responders reported higher rates of compensation differences (*p* = 0.002), in addition to higher rates of discrimination in job promotion (*p* = 0.006), compared to male respondents. Without specific job description or academic rank comparison, it is difficult to make a firm conclusion. However, transparency about salary is not universal; therefore, one could also argue that differences in compensation could be even higher than reported. We fully support equal pay for same rank same job descriptions for everyone. We propose every institution should make the best possible effort to be transparent about compensation.

To our knowledge, this is the first report of self-reported discrimination in the field of LT. Gender, race, and country of origin were amongst the highest categories that ILTS members felt the most disparity. A 34.7% reported rate of disparity amongst our members is alarming and requires immediate attention by transplant stakeholders. While we are aware that change in the culture of medicine or the field of liver transplant takes time, and that it is clearly subject to local regulatory rules, we propose the following as possible solutions followed by international societies: 1- Promote underrepresented groups and female providers in academic and clinical work, which would require institutional support; 2- Create individual mentorship programs within institutions, starting at the trainee and staff level and extending to include junior faculty 3- Advocate for actual paternity leave policies 4- Create a work environment of caring about equity, diversity and inclusion. This could start with local dedicated EDI committees within the division, initially through volunteers and eventually extending to participation of all division members. 5-Professional societies prioritize or perhaps use 50-50 quota (i.e., 50% men, 50% women and other minority groups) that is inclusive of women, underrepresented gender and professionals from various countries of origin at meetings, sessions, chair positions, presenter selection or process selections.

Limitations of our report include the following: 1) a low response rate of 15%. This could certainly cause bias, for instance, we might have received more answers from members with negative experiences. Yet, our society distribution is very similar in major characteristics such as gender, age, and specialty distribution to that which responded to the survey; 2) a relatively higher leadership representation responded in compared to that of the ILTS society. This could be interpreted different ways. On the one hand, this may mean a high number of participants who were trained in the remote past i.e., at an “era” that did not take EDI issues into consideration and, hence, a personal experience that may not be representative of today’s LT world. In other words, there could be an “era effect” in our results and potential era bias. On the other hand, it may mean the contrary, a high number of participants that have eventually climb the ladder but want to express the difficulties in getting there; 3) Lastly, it is difficult to actually compare compensation responses; as, we do not have details of job description: i.e. full time job, academic rank comparison, or country specific variations.

## Conclusion

In conclusion, we, herein, report the first international survey among liver transplant providers regarding disparity and female leadership. Our survey suggests that liver transplant providers may experience discrimination based on gender or race, lack of mentorship or support for discriminatory actions and very low rates of female representation in LT leadership positions, the lowest being in liver transplant surgery. In addition, we identified higher rates of overall discrimination, discrimination in job promotion as well as compensation differences reported by female LT providers compared to male respondents. Identifying the reality is the first step to mitigate these issues. As the ILTS EDI-committee, we propose some action items. Furthermore, we at the ILTS will continue to work towards creating various task forces and work groups to dive deep in these disparity issues.

## Data Availability

The original contributions presented in the study are included in the article/Supplementary Material, further inquiries can be directed to the corresponding author.

## Footnotes

1
https://www.ilo.org/global/topics/care-economy/WCMS_838653/lang--en/index.htm

## Abbreviations

ASTS, American society of transplant surgeons; AST, American society of transplantation; EDI, equality, diversity and inclusion; ILTS, The International liver transplant society; LT, liver transplantation; ILO, International labor office.
