# Supplementary material for: Gender and Racial Disparity Among Liver Transplantation Professionals: Report of a Global Survey
Source: Transpl Int. 2022 Aug 16;35:10506. doi: 10.3389/ti.2022.10506 (PMC9426639; doi:10.3389/ti.2022.10506)
Supplement: Supplementary file 1 [file Table1.DOCX]

**Supporting Information:**

**Supplementary Table: Baseline characteristics from survey respondents and ILTS members:**

|  | **Survey**  **(N=199)** | **ILTS members***  **(N=787 of 1312)** | **P value** |
| --- | --- | --- | --- |
| Age  - <30 years  -Between 30-39 years  -Betwen 40-49 years  -Betwen 50-59 years  -Betwen 60-69 years  ->70 years | 2 (1%)  51 (25.6%)  79 (39.7%)  45 (22.6%)  20 (10.1%)  2 (1%) | 0%  <40 yrs:31%  40-49 yrs:35%  50-59 yrs:20%  11%  3% | 0.2651 |
| Gender  -Female  -Male  -Other | 81(40.7%)  118 (59.3%)  0(0%) | 31%*  69%*  na | 0.093 |
| Geographic location  -North American  -Europe  -Asia  -Others | 42%  36%  14%  8.5% | 44%  21%  24%  11% | <0.001 |
| Job position  -Surgery  -Hepatology  -Anesthesia or Medical care  -Others | 91 (45.7%)  55 (27.6%)  53 (26.6%)  25 (12.5%) | 46%  15%  28%  11% | <0.001 |

*Data on ILTS gender is only available for 60% of membership
