# Supplementary material for: Gender and Racial Disparity Among Liver Transplantation Professionals: Report of a Global Survey
Source: Transpl Int. 2022 Aug 16;35:10506. doi: 10.3389/ti.2022.10506 (PMC9426639; doi:10.3389/ti.2022.10506)
Supplement: Supplementary file 2 [file DataSheet1.docx]

**Supplement 1**

**Survey Questions**

Q1: **How old are you?** under 30 years, between 30-39 years, between 40-49 years, between 50-59 years, between 60-69 years, over 70 years

Q2: **What is your gender?** women, men, other

Q3:**What is your ethnicity?** Caucasian, Asian, Hispanic, or Latino, African or African American

Q4: **In which country do you perform your liver transplant related job?**

Q5: **In which type of working place?** Academic hospitals, Private Hospitals, Government hospitals, Other (please specify)

Q6: **For how long have you been in practice since fellowship**? 5 or less years, 5-10 years, more than 10 years

Q7: **What is your current job position?** Chief, Medical Doctor, Medical Doctor in Training, Researcher, Others (please specify)

Q8: **What is your main specialty?** Surgery, Hepatology, Anesthesia or Intensive Care, Pediatric medicine, other activities (please specify)

Q9: **What is the sex of the person in the leadership position for your department or work unit?** Male, Female

Q10: **How many LT does your institution perform each year?** Less than 50, Between 50-100, Between 100-200, More than 200

Q11: **Have you ever experienced any discrimination during your training or in your current position?** Yes, No

Q12: **If you answered yes to the previous question, could you indicate the presumed basis of that discrimination**: Gender, Religion, Race, Country of origin, Sexual orientation, Prefer not to answer, Others

Q13: **Have you ever felt at disadvantage for a job promotion because of possible negative discrimination against you?** Yes, No

Q14**: If you are a parent, was your institution supportive of your pregnancy or parenteral leave?** Yes, No, Neither supportive nor unsupportive,

Q15: **Are you aware of differences in compensations (salary, bonus, incentive payments, research stipends, honoraria and distribution of profits to employees) between women and men in your workplace?** Yes, No

Q16: **Have you had the possibility to work with or receive support from a mentor during your training career in relation to discrimination issues?** Always, Usually, Sometimes, Rarely, Never

Q18: **Have you ever felt at disadvantage in relation to your country of origin or language skills in terms of participating in ILTS meetings or leading collaborative projects?** Yes, No

Q19: **Would you be willing to participate as a speaker, moderator or in the SIG of your interest in future ILTS meetings?** Yes, No
